# Impact of Resident Involvement on 30-Day Postoperative Outcomes in Orthopedic Shoulder Surgery

**DOI:** 10.1155/2024/1550500

**Published:** 2024-03-31

**Authors:** Aaron J. Marcel, Richard S. Feinn, Karen M. Myrick

**Affiliations:** ^1^Frank H. Netter MD School of Medicine at Quinnipiac University, North Haven, Connecticut, USA; ^2^University of Saint Joseph, West Hartford, CT, USA

## Abstract

The literature concerning resident involvement in shoulder surgery is limited. The purpose of this study was to examine whether resident involvement across all orthopedic shoulder surgeries is associated with adverse 30-day outcomes. Utilizing the American College of Surgeons National Surgical Quality Improvement Program database, patients who underwent shoulder surgery with or without a resident present were analyzed. Independent *t*-test and chi-square or Fischer's exact test were used appropriately. A logistic regression model was used to calculate adjusted odds ratios. This study examined 5,648 patients: 3,455 patients in the “Attending alone” group and 2,193 in the “Attending and resident in the operating room” group. Resident presence in the operating room was not associated with increased complications, except for bleeding transfusions (OR 1.71, CI 1.32–2.21, *P* ≤ 0.001). This study demonstrates that resident involvement in orthopedic shoulder surgery does not present an increased risk for 30-day complications when compared to surgeries performed with the attending surgeon alone.

## 1. Introduction

Orthopedic surgery residency is held to the highest standard of knowledge and expertise. Utilizing a structure focused on graduated responsibility and procedural competency, orthopedic residents are trained to be proficient in the prevention, diagnosis, and treatment of musculoskeletal injuries and diseases [1]. Resident education, however, is not without its challenges. In a survey of 12 program directors or chairs, Laporte et al. highlighted the major issues in orthopedic resident training as the following: loss of professionalism, too much emphasis on procedures, lack of clinical experience, and external oversight [2]. These issues may leave residents unprepared when it comes to accepting the full responsibilities of an attending physician. Furthermore, residency program requirements are evolving, with residents working less hours to avoid burnout while also handling increasingly complex cases [2–5]. In an editorial commentary for *Arthroscopy*: *The Journal of Arthroscopic and Related Surgery*, Nikhil Verma noted that attending physicians struggle with balancing patient care, efficiency, and resident education [6]. With the increasing complexity of orthopedic surgery, it is becoming more difficult to implement the principles of graduated responsibility, limiting the experience available for resident training.

Much of the concern with resident involvement in surgery stems from the patient's perspective. In a 2001 study by Cowles et al., one-third of general surgery patients did not want residents involved in their surgery, while majority of the remaining participants expressed that they would only want residents involved in minor procedures [7]. In an observational study, Versluis and van der Linden examined patient attitudes towards resident involvement in gynecological surgery and found that patients have less confidence in less experienced residents even when supervised by an attending, as well as less confidence in all residents operating without supervision (*P* < 0.001) [8]. Looking at orthopedic surgery, Nahhas et al. found that 94.1% of survey respondents believed that residents should perform surgery as part of their training, yet 39.7% were not satisfied with a second-year resident assisting in their surgery. In addition, 92.2% felt that resident involvement should be disclosed to the patient [9].

Despite the challenges facing orthopedic surgery residents and patient concerns toward resident involvement in surgery, several studies have shown that resident participation in orthopedic surgery does not significantly impact a number of surgical and medical outcomes [3–6, 10–33]. While previous studies have documented the outcomes of resident involvement in hip [11, 15, 22], knee [12, 27, 30], spine [10, 21], and trauma surgery [34], few have looked at the outcomes of shoulder surgery with resident involvement. Of these studies, only bicep tenodesis [20], shoulder stabilization [35], shoulder arthroscopy [3, 13, 18], and total shoulder arthroplasty (TSA) [19, 36] have been examined. In reviewing the available orthopedic literature, no study has investigated the impact of resident involvement across all types of shoulder surgeries.

The purpose of this study was to examine the impact of resident involvement in all orthopedic shoulder surgeries using The American College of Surgeons National Surgical Quality Improvement (ACS-NSQIP) database.

## 2. Methods

### 2.1. Data Source

The American College of Surgeons National Surgical Quality Improvement Program (ACS-NSQIP) is a national, validated, risk-adjusted, and prospectively maintained surgical outcomes registry that contains more than 240 clinical variables including preoperative patient characteristics, intraoperative variables, and 30-day postoperative outcomes. The NSQIP database allows for high-powered, retrospective analyses over a wide array of surgical procedures using Current Procedural Terminology (CPT) codes. In addition, the NSQIP database has been deemed more accurate than other surgical complications databases and those based on insurance claims, as well as surgical mortality and morbidity conferences [37–39].

### 2.2. Data Collection

Using CPT codes 23000–23929, a retrospective review using the NSQIP database was conducted to identify all cases of orthopedic shoulder surgery between 2010 and 2012. Shoulder surgery cases based on CPT codes included incision procedures, excision procedures, introduction or removal procedures, repairs, revisions, reconstructions, fractures, dislocations, manipulations, arthrodesis, and amputations. This resulted in 5,648 cases for analysis. These cases were divided and analyzed based on resident presence in the operating room. The “ATTEND” variable was used to determine the two cohorts: “Attending alone” and “Attending and resident in operating room.”

Patient demographics were defined by age, body mass index (BMI), sex, race, and ethnicity. The comorbidities analyzed included >10% body weight loss in <6 months, ascites, bleeding disorders, congestive heart failure (CHF), chronic obstructive pulmonary disease (COPD), current smoker, diabetes, currently on dialysis, disseminated cancer, dyspnea, alcoholism defined as drinking >2 alcoholic drinks per day, functional status, hypertension, rest pain/gangrene, and exogenous steroid use. Operative characteristics included American Society of Anesthesiologists (ASA) classification, emergency case, blood transfusions >4 U of packed red blood cells (PRBC's), and total operation time.

Outcomes were divided into medical complications, surgical complications, and other complications. Medical complications consisted of acute renal failure, bleeding transfusion, cardiac arrest, cerebrovascular attack (CVA)/stroke, deep vein thrombosis/thrombophlebitis, myocardial infarction, peripheral nerve injury, pneumonia, progressive renal insufficiency, pulmonary embolism, sepsis, septic shock, unplanned intubation, urinary tract infection, and being on a ventilator for >48 hours. Surgical complications included deep surgical site infections (SSI), organ/space SSI, superficial SSI, wound dehiscence, and wound occurrences. Other complications included any readmission, inpatient stay >30 days, return to the operating room, unplanned readmission, and unplanned reoperation.

### 2.3. Statistical Analysis

Demographics, comorbidities, operative characteristics, and outcomes were analyzed using descriptive and comparative statistics. For continuous variables, an independent samples *t*-test was used. For categorical variables, Pearson's chi-square or Fischer's exact test were used appropriately. Propensity scores, categorized into quintiles, were used for risk adjustment to control for baseline differences in the cohort prior to surgery. The logistic regression model used the propensity score quintile and attending presence as the predictor variables to calculate the adjusted odds ratio for outcomes. All statistical analysis was performed using the Statistical Package for Social Sciences (SPSS) 26, and statistical significance was set at an alpha level of 0.05.

## 3. Results

Review of the NSQIP database for all orthopedic shoulder surgeries yielded 5,648 cases. Dividing these cases based on resident presence resulted in 3,455 cases for “Attending alone” and 2,193 cases for “Attending and resident in operating room” groups.

Patient demographics between the two cohorts differed significantly in race and ethnicity. Residents were less likely to be present for operations on patients who were White (Attending alone 82.5% vs Resident present 76.3%), Native American or Alaskan (Attending alone 0.7% vs Resident present 0.4%), and Native Hawaiian or Pacific Islander (Attending alone 0.2% vs Resident present 0.0%), but more likely to be present for patients who were Black (Attending alone 3.3% vs Resident present 4.8%) or “Other” race (Attending alone 12.6% vs Resident present 17.7%) (*P* ≤ 0.001), as well as patients who were non-Hispanic (Attending alone 93.5% vs Resident present 97.6%, *P* < 0.001). Regarding comorbidities, residents were significantly more likely to be present in the operating room for patients who had a >10% loss in body weight in less than 6 months (*P*=0.05), bleeding disorders (*P*=0.045), congestive heart failure (CHF) (*P*=0.021), disseminated cancer (*P* < 0.001), partially dependent functional status (*P* < 0.001), and history of steroid use for chronic conditions (*P* < 0.001), but significantly less likely to be present for patients who had dyspnea (*P*=0.04) (Table 1). There were no demographic differences between groups after stratification of propensity scores. Further, residents were more likely to be present in cases that were classified as emergencies (*P* < 0.001) and resident involvement also resulted in significantly longer operative times (92.3 ± 52.3 vs 111.2 ± 70.1, *P* < 0.001). Both remained significant after propensity score stratification (Emergency Case: *P* ≤ 0.001, Total Operation Time: *P* < 0.001) (Table 2).

In the analysis of operative outcomes, with propensity score adjustment in a logistic regression model, resident presence was associated with a significantly increased number of bleeding transfusions (OR 1.71, CI = 1.32–2.21, *P* < 0.001). Resident presence in the operating room was not associated with any other medical, surgical, or other complication (Table 3).

## 4. Discussion

Using the nationwide, high-quality NSQIP database from the American College of Surgeons, this study is the first to analyze the impact of resident involvement in all orthopedic shoulder surgeries. Analysis of 5,648 patients demonstrated that resident presence in the operating room during orthopedic shoulder surgery does not significantly impact overall 30-day outcomes. This agrees with previous studies across various divisions of orthopedic surgery, including shoulder, knee, hip, spine, and trauma [3–6, 10–33]. Additionally, this study agrees with the well-documented conclusion that resident presence increases operation time [3, 4, 13, 15–17, 21, 22, 25, 29, 31, 33, 35].

Few studies have examined resident impact on postoperative outcomes in orthopedic shoulder surgery. In a similar study examining resident involvement in shoulder arthroscopy, Basques et al. found the overall rate of adverse events to be 1.09%. Interestingly, and in disagreement with other studies and the present study, Basques et al. found no increase in operation time with resident participation [18]. Also, examining shoulder arthroscopy, Gulbrandsen et al. used a propensity-matched analysis to demonstrate that there was no difference in the overall rate of 30-day complications in the resident versus nonresident group (*P*=0.576) [13]. Jovan et al. illustrated no significant increase in 30-day postoperative outcomes when examining resident involvement in shoulder stabilization surgery [35]. Pugely et al. conducted a retrospective study investigating resident impact on short-term outcomes in various orthopedic surgeries. Grouping basic arthroscopy of the shoulder and knee, as well as advanced arthroscopy in rotator cuff repairs and anterior cruciate ligament (ACL) reconstructions, the authors found no difference in mortality (basic arthroscopy OR = 3.41, CI = 0.75–15.46, *P* ≥ 0.05; advanced arthroscopy OR = N/A) or any morbidity (basic arthroscopy OR = 0.84, CI = 0.60–1.17, *P* ≥ 0.05; advanced arthroscopy OR = 0.78, CI = 0.44–1.34. *P* ≥ 0.05) [3]. Looking at postoperative complications after TSA, Cvetanovich et al. found an insignificant decrease in overall rate of 30-day complications when a resident was involved (2.60%) versus attending only (3.91%) (*P*=0.173) [19]. The “July Effect” is an assumed risk to patients that correlates with new medical school graduates entering residency training. A 2017 study by Rao et al. found no evidence for the “July Effect” in TSA, demonstrating that resident involvement did not increase risk of complications [36].

The present study demonstrates that resident presence in orthopedic shoulder surgeries does not have a significant impact in short-term postoperative outcomes other than an increase in blood transfusions in the resident group (OR 1.71, *P* ≤ 0.001). Due to the limitations of the NSQIP database, it is not possible to attribute the need for increased blood transfusions in the resident group to any particular cause. However, this outcome is likely multifactorial. Increased need for blood transfusions may be attributed to the fact that that residents were also more likely to be present in emergency cases (*P* ≤ 0.001), longer operations (*P* ≤ 0.001), and for operations on patients who have bleeding disorders (*P*=0.045). Additionally, residents may be more involved in operations of increased complexity, such as TSA and reverse TSA (rTSA), as attendings are likely to need assistance. The need for blood transfusions following shoulder arthroplasty has a reported range of 3.9%–43% [40–46]. Further, postoperative hematoma formation is one of the most common complications of rTSA [47, 48]. For a variety of potential reasons, the findings in this study highlight the need for careful consideration of blood management in procedures in which a resident is involved. However, minimizing blood loss is an important consideration for any surgical procedure and focused attention should still be given to potential blood loss in all operations, regardless of the personnel involved. This study otherwise illustrates that resident involvement in orthopedic shoulder surgery presents an overall low risk regarding 30-day postoperative outcomes.

Much of the discussion regarding resident involvement in surgical procedures is based on patient concern [7–9]. This study provides reassurance to patients planning to undergo orthopedic shoulder surgery who are concerned about resident involvement in their surgery. This study, among other studies demonstrating similar findings, can be a counseling aid for physicians faced with apprehensive patients. Additionally, the findings of this paper argue in favor of resident participation in shoulder surgery and illustrate that orthopedic residency training is accomplishing what it aims to in creating competent surgeons.

The present study is not without limitations, several of which are inherent to the NSQIP database. First, NSQIP only records 30-day outcomes. Consequently, complications occurring more than 30 days after the index operation could not be determined, and the present study may underrepresent the overall mortality and morbidity regarding resident involvement in orthopedic shoulder surgery. Second, postgraduate year for residents was underreported in the NSQIP database, resulting in exclusion of the PGY variable in this study. This could introduce bias if PGY was positively or negatively skewed. Third, NSQIP does not determine the degree of resident involvement in patient care. There could be other confounders during perioperative care which could alter the results of this study. Also, the NSQIP database lacks data on the involvement of other healthcare professionals (i.e., nurses, personal care assistants, pharmacists, etc.), who could potentially affect patient outcomes. Additionally, we examined all shoulder surgeries under a single cohort in order to analyze a broad category of residency training. This study is, therefore, unable to assess differences among specific procedures. Few other studies have assessed surgical subtypes, and this may continue to be an area of focus for future research. Lastly, the present study is retrospective and, despite using propensity scores to adjust for potential confounders, other uncontrolled variables may differ between groups and introduce bias. The limitations of this study coincide with those of similar studies which have examined resident involvement in various surgical specialties.

## 5. Conclusion

Focused on the ideal of graduated responsibility, it is imperative that residents in orthopedic surgery receive high-quality, rigorous training. Residents are expected to be excellent diagnosticians and procedurally competent physicians by the time they are ready to move on to the next tier of their career. Still, many people in the general public have expressed concern with resident involvement in their surgeries. This study compared 30-day outcomes between “Attending alone” and “Attending and resident in the operating room” groups in all orthopedic shoulder surgeries. Using a propensity score adjusted logistic regression, no association was found between resident involvement and increased occurrence of all 30-day complications, except for bleeding transfusions (*P* ≤ 0.001). The results of this study demonstrate an overall low risk for 30-day complications from resident involvement in all orthopedic shoulder surgeries. These findings may be used as an educational tool to counsel patients who are concerned about resident involvement in their orthopedic shoulder surgery.

## Figures and Tables

**Table 1:** Demographic data and comorbidities in 5,648 patients treated with orthopedic shoulder surgery in 2010–2012, categorized by resident presence.

Variable	Resident present				*P* value
	No (3455 patients)		Yes (2193 patients)		
	Value	%	Value	%	
Demographic					
Age in yrs, mean ± SD	59.9 ± 15.6		59.4 ± 16.9		0.229
BMI in kg/m^2^, mean ± SD	29.6 ± 8.1		28.8 ± 8.2		0.958
Sex					0.444
Male	1770	51.3	1042	47.6	
Female	1680	48.7	1146	52.4	
Race^*∗*^					<0.001
Asian	24	0.7	16	0.7	
Black	115	3.3	106	4.8	
Native American or Alaskan	25	0.7	9	0.4	
Native Hawaiian or Pacific Islander	6	0.2	0	0.0	
White	2851	82.5	1673	76.3	
Other	434	12.6	389	17.7	
Ethnicity^*∗*^					<0.001
Hispanic	224	6.5	52	2.4	
Non-Hispanic	3231	93.5	2141	97.6	
Comorbidities					
>10% loss body weight in <6 mo^*∗*^	5	0.1	9	0.4	0.050
Ascites	0	0.0	2	0.1	0.076
Bleeding disorders^*∗*^	88	2.5	76	3.5	0.045
CHF^*∗*^	3	0.1	8	0.4	0.021
COPD	159	4.6	89	4.1	0.331
Current smoker	601	17.4	396	18.1	0.525
Diabetes	512	14.8	310	14.1	0.478
Dialysis	14	0.4	11	0.5	0.595
Disseminated cancer^*∗*^	13	0.4	36	1.6	<0.001
Dyspnea^*∗*^	250	7.2	128	5.8	0.040
EtOH >2 drinks/day	126	3.6	70	3.2	0.363
Functional status (partially dependent)^*∗*^	96	2.8	107	4.9	<0.001
Hypertension	1759	50.9	1101	50.2	0.605
Rest pain/Gangrene	3	0.1	2	0.1	0.957
Steroid use^*∗*^	86	2.5	94	4.3	<0.001

BMI = body mass index; CHF = congestive heart failure; COPD = chronic obstructive pulmonary disease. ^*∗*^Denotes significant difference between cohorts (*P* < 0.05).

**Table 2:** Operative characteristics in 5,648 patients treated with shoulder surgery in 2010–2012, categorized by resident presence.

Operative variable	Resident present				*P* value
	No (3455 patients)		Yes (2193 patients)		
	Value	%	Value	%	
ASA classification					0.247
Class 1, no disturbance	395	11.5	226	10.3	
Class 2, mild disturbance	1708	49.5	1147	52.3	
Class 3, severe disturbance	1256	36.4	770	35.1	
Class 4, life threatening disturbance	89	2.6	50	2.3	
Class 5, moribund	1	<0.1	0	0.0	
Emergency case^*∗*^	102	3.0	110	5.0	<0.001
Transfusion >4 U PRBC's 72 hr preoperative	16	0.5	18	0.8	0.090
Total operation time in min, mean ± SD^*∗*^	92.3 ± 52.3		111.3 ± 70.1		<0.001

ASA = American Society of Anesthesiologists, PRBC's = packed red blood cells. ^*∗*^Denotes significant difference between cohorts (*P* < 0.05).

**Table 3:** Postoperative outcomes in 5,648 patients treated with shoulder surgery in 2010–2012, categorized by resident presence.

Outcome	Resident present				OR	CI	*P* value
	No (3455 patients)		Yes (2193 patients)				
	Value	%	Value	%			
Medical complications							
Acute renal failure	2	0.1	1	<0.01	1.10	0.10–12.33	0.939
Bleeding transfusion^*∗*^	120	3.5	132	6.0	1.71	1.32–2.21	<0.001
Cardiac arrest	5	0.1	0	0.0	0.00	0.00	0.986
CVA/Stroke	5	0.1	2	0.1	0.64	0.12–3.34	0.599
DVT/thrombophlebitis	17	0.5	9	0.4	0.77	0.34–1.75	0.534
Myocardial infarction	5	0.1	3	0.1	0.80	1.88–3.39	0.762
Peripheral nerve injury	4	0.1	6	0.3	2.44	0.68–8.70	0.169
Pneumonia	19	0.5	11	0.5	0.88	0.42–1.87	0.742
Progressive renal insufficiency	2	0.1	2	0.1	1.48	0.20–10.76	0.701
Pulmonary embolism	9	0.3	8	0.4	1.42	0.54–3.73	0.475
Sepsis	9	0.3	7	0.3	1.04	0.38–2.83	0.936
Septic shock	2	0.1	2	0.1	1.59	0.22–11.48	0.647
Unplanned intubation	9	0.3	2	0.1	0.36	0.08–1.68	0.194
Urinary tract infection	26	0.8	15	0.7	0.94	0.49–1.79	0.845
Ventilator >48 hrs	5	0.1	2	0.1	0.56	0.11–2.93	0.489
Surgical complications							
Deep SSI	8	0.2	6	0.3	0.98	0.34–2.86	0.976
Organ/space SSI	11	0.3	5	0.2	0.68	0.23–1.97	0.478
Superficial SSI	10	0.3	9	0.4	1.37	0.55–3.41	0.493
Wound dehiscence	5	0.1	4	0.2	1.27	0.34–4.80	0.723
Wound occurrences	10	0.3	9	0.4	1.37	0.55–3.41	0.493
Other complications							
Any readmission	28	1.4	30	2.1	1.44	0.85–2.44	0.172
In hospital >30 days	3	0.2	0	0	0.00	0.00	0.988
Return to OR	49	1.4	28	1.3	0.83	0.51–1.32	0.426
Unplanned readmission	26	1.3	26	1.8	1.32	0.76–2.30	0.324
Unplanned reoperation	10	0.5	8	0.6	1.07	0.42–2.73	0.895

CVA = cerebrovascular attack, DVT = deep vein thrombosis, SSI = surgical site infection. ^*∗*^Denotes significant difference between cohorts (*P* < 0.05).

## Data Availability

The data that support these findings are housed with the American College of Surgeons. Data are available in de-identified fashion to participants of the NSQIP Transplant Program.

## Additional Points

*Statement of Clinical Significance*. This manuscript highlights an important issue in the patient-physician relationship and may be used as a tool to build trust and counsel patients who may be hesitant in having residents involved in their care.
